# Nanopore Sequencing Using the Full-Length 16S rRNA Gene for Detection of Blood-Borne Bacteria in Dogs Reveals a Novel Species of Hemotropic Mycoplasma

**DOI:** 10.1128/spectrum.03088-22

**Published:** 2022-10-17

**Authors:** Lucas G. Huggins, Vito Colella, Ushani Atapattu, Anson V. Koehler, Rebecca J. Traub

**Affiliations:** a Faculty of Veterinary and Agricultural Sciences, University of Melbourne, Parkville, Victoria, Australia; University of Saskatchewan

**Keywords:** hemotropic mycoplasmas, nanopore, vector-borne pathogens, MinION sequencing, canines, 16S rRNA, *Anaplasma*, *Bartonella*, *Ehrlichia*, Oxford Nanopore Technologies, blood microbiome, long-read sequencing

## Abstract

Dogs across the globe are afflicted by diverse blood- and vector-borne bacteria (VBB), many of which cause severe disease and can be fatal. Diagnosis of VBB infections can be challenging due to the low concentration of bacteria in the blood, the frequent occurrence of coinfections, and the wide range of known, emerging, and potentially novel VBB species encounterable. Therefore, there is a need for diagnostics that address these challenges by being both sensitive and capable of detecting all VBB simultaneously. We detail the first employment of a nanopore-based sequencing methodology conducted on the Oxford Nanopore Technologies (ONT) MinION device to accurately elucidate the “hemobacteriome” from canine blood through sequencing of the full-length 16S rRNA gene. We detected a diverse range of important canine VBB, including Ehrlichia canis, Anaplasma platys, Mycoplasma haemocanis, Bartonella clarridgeiae, “*Candidatus* Mycoplasma haematoparvum”, a novel species of hemotropic mycoplasma, and *Wolbachia* endosymbionts of filarial worms, indicative of filariasis. Our nanopore-based protocol was equivalent in sensitivity to both quantitative PCR (qPCR) and Illumina sequencing when benchmarked against these methods, achieving high agreement as defined by the kappa statistics (*k *> 0.81) for three key VBB. Utilizing the ability of the ONT’ MinION device to sequence long read lengths provides an excellent alternative diagnostic method by which the hemobacteriome can be accurately characterized to the species level in a way previously unachievable using short reads. We envision our method to be translatable to multiple contexts, such as the detection of VBB in other vertebrate hosts, including humans, while the small size of the MinION device is highly amenable to field use.

**IMPORTANCE** Blood- and vector-borne bacteria (VBB) can cause severe pathology and even be lethal for dogs in many regions across the globe. Accurate characterization of all the bacterial pathogens infecting a canine host is critical, as coinfections are common and emerging and novel pathogens that may go undetected by traditional diagnostics frequently arise. Deep sequencing using devices from Oxford Nanopore Technologies (ONT) provides a solution, as the long read lengths achievable provide species-level taxonomic identification of pathogens that previous short-read technologies could not accomplish. We developed a protocol using ONT’ MinION sequencer to accurately detect and classify a wide spectrum of VBB from canine blood at a sensitivity comparable to that of regularly used diagnostics, such as qPCR. This protocol demonstrates great potential for use in biosurveillance and biosecurity operations for the detection of VBB in a range of vertebrate hosts, while the MinION sequencer’s portability allows this method to be used easily in the field.

## INTRODUCTION

Blood-borne bacterial infections of dogs represent a diverse spectrum of organisms capable of producing a range of pathologies, from those that are relatively benign through to life-threatening conditions (1, 2). Among these, vector-borne bacteria (VBB) affect dogs around the globe, with their transmission facilitated by the bites of hematophagous arthropods, such as ticks, fleas, and flies (3, 4). Globally abundant and important ectoparasites, such as the brown dog tick, have permitted the continued dissemination of potentially deadly VBB like Ehrlichia canis and Rickettsia rickettsii, the latter of which is also an important zoonotic agent. Other VBB, such as Anaplasma platys, *Bartonella* spp., *Borrelia* spp., and hemotropic mycoplasmas, can all cause morbidity in dogs, exacerbating clinical signs particularly in those that are immunocompromised (2, 3, 5–9). Moreover, brown dog ticks can act as a vector for multiple canine VBB, and if left untreated, these infections may last the animal’s life span (5, 10). Hence, coinfection with VBB is not uncommon, making accurate diagnosis even more complex (11). To alleviate these issues, diagnostic methods capable of accurately detecting and characterizing VBB coinfections in a time-effective manner are essential, particularly in regions where canine pathogen diversity is high, such as the tropics (12, 13).

Molecular techniques, including conventional PCR (cPCR) and real-time PCR, are increasingly used for the detection and clinical diagnosis of canine VBB, owing to their superior sensitivity over traditional methods, such as examination of blood smears and bacterial cultures (12, 14–17). More recently, next-generation sequencing (NGS)-based methods, capable of detecting the complete diversity of bacteria from a sample simultaneously, have been adopted as alternatives for VBB detection (18–21). These techniques can be used to amplify and sequence either short stretches, or the full length, of universal bacterial genetic targets, such as the 16S rRNA gene, which is species diagnostic for most bacteria if its full length is used (22). Sequencing of either an informative hypervariable region(s) or the full length of this gene for all bacteria present in a sample forms a 16S rRNA gene “metabarcode,” providing information on the presence of known or putative pathogens, including the presence of coinfections (12, 21, 23).

NGS using the Illumina platform has been one of the most widely adopted methods for bacterial community analysis to date, due to the high depth of sequencing achievable and excellent raw read accuracy (24, 25). Nonetheless, such methods are hindered by a maximum sequence length of about 500 bp and long library preparation and sequencing times (19, 24, 25). The arrival of third-generation sequencing methods such as nanopore-based sequencing by Oxford Nanopore Technologies (ONT) has obviated many of these limitations, including the ability to provide theoretically uncapped sequencing lengths with usable results obtained in as little as 5 min (19, 26, 27). Although the native single-read error rate of nanopore-based sequencing is typically much higher than that of other NGS methods, this can be corrected for by the use of bioinformatic approaches (24, 28, 29).

For microbiome analysis, the longer read lengths attainable through ONT platforms permit sequencing of the entire 16S rRNA gene or the full *rrn* operon, providing greatly improved taxonomic resolution (25, 30, 31). Such species- or even strain-level characterization is of crucial importance for veterinarians and clinicians, given the significant interspecific differences in pathogenicity and treatment approaches that exist between pathogens (31, 32). Additionally, the small size of ONT sequencers, such as the MinION, makes them highly portable and amenable to use in mobile laboratories, whereby sequencing and diagnosis can be conducted in the field or at the point of care (26, 27, 33, 34).

Taking this into consideration we, for the first time, optimized and validated the use of nanopore-based metabarcoding of blood-borne bacteria of dogs by using the MinION device and explored whether it could be used as a feasible method for canine VBB diagnosis. To achieve this, we tested a modified ONT 16S rRNA gene amplification and sequencing protocol on a diverse range of VBB-positive controls while also benchmarking our method against a highly sensitive and specific multiplex quantitative PCR (qPCR) as well as an Illumina-based NGS diagnostic (14, 21). Methodological comparison was conducted with a bank of 100 canine blood DNA samples from Cambodia, a country that has been found to have a high biodiversity of VBB at an alarming prevalence in many regions (12, 35).

## RESULTS

### Nanopore sequencing of the 16S rRNA gene methodological validation.

To check for PCR amplification biases generated by the 27F and 1429R 16S rRNA gene-targeting primers, a ZymoBIOMICS microbial community DNA standard was run, and the results were assessed (Table 1). All the expected bacterial species were detected and correctly classified, but relative abundances were different from those of the original manufactured composition of this mock community. Species such as Staphylococcus aureus and Bacillus subtilis were substantially overrepresented, while Pseudomonas aeruginosa was underrepresented by about 10% (Table 1).

**TABLE 1:** Performance of the 27F and 1429R 16S rRNA gene-targeting primers on the ZymoBIOMICS microbial community DNA standard positive control^*a*^

Species	Expected abundance (%)	NanoCLUST abundance (%)
Bacillus subtilis	12	16
Enterococcus faecalis	12	9
Escherichia coli	12	10
Lactobacillus fermentum	12	9
Listeria monocytogenes	12	15
Pseudomonas aeruginosa	12	2
Salmonella enterica	12	14
Staphylococcus aureus	12	18
Other	4	7

a The table shows expected abundance as detailed by ZymoBIOMICS, which was identified after processing and taxonomic classification by the NanoCLUST pipeline.

A large array of VBB were detectable from canine blood, cell culture, and spots on slides coated with cultured organisms, with accurate formation of consensus sequences that consistently had more than 99.3% identity to representative sequences in GenBank (Table 2). Correct classification was observed even between close taxonomic relatives such as the *Rickettsia* species tested, while coinfections of between two and three VBB were also identified (Table 2). Positive-control samples from cell culture and hemolymph typically had higher read counts than those from canine blood samples, likely due to blood being a low-biomass sample type for bacteria (36). Concentrations of DNA from positive controls (cell culture and spots on slides coated with cultured organisms) ranged from 0.05 ng/μL to 4.45 ng/μL, while concentrations of whole-blood DNA from Cambodian dogs were between 0.44 ng/μL and 65.9 ng/μL. Dog blood samples found positive for microfilaria via microscopy and confirmed via cPCR returned many 16S rRNA gene reads from *Wolbachia* endosymbionts identified from the relevant filarial worm species (Table 2).

**TABLE 2:** Performance of our modified 16S rRNA gene nanopore sequencing protocol and NanoCLUST-based classification on confirmed bacterium- and filarial worm-positive controls^*a*^

Pathogen(s) present	Sample type	NanoCLUST classification	NCBI accession no.	Length (bp)	Identity (%)	No. of reads
Bacterial						
Anaplasma phagocytophilum	Slides coated with cultured organisms	Anaplasma phagocytophilum	CP006618.1	1,422	99.93	3,297
Anaplasma platys	Canine blood	Anaplasma platys	CP046391.1	1,422	100	1,007
Bartonella henselae	Flea hemolymph	Bartonella henselae	CP020742.1	1,446	100	35,889
Borrelia burgdorferi	Cell culture	Borrelia burgdorferi	CP031412.1	1,430	100	46,843
“*Candidatus* Mycoplasma haematoparvum”	Canine blood	“*Candidatus* Mycoplasma haematoparvum”	AY383241.1	1,417	99.79	1,967
Ehrlichia canis	Canine blood	Ehrlichia canis	CP025749.1	1,420	100	5,103
Ehrlichia chaffeensis	Slides coated with cultured organisms	Ehrlichia chaffeensis	NR_074500.2	1,420	100	2,129
Leptospira biflexa	Cell culture	Leptospira biflexa serovar Patoc	CP000786.1	1,417	100	34,669
Mycoplasma haemocanis	Canine blood	Mycoplasma haemocanis	CP003199.1	1,354	99.79	217
Orientia tsutsugamushi	Cell culture	Orientia tsutsugamushi	LS398550.1	1,417	100	67
Rickettsia australis	Cell culture	Rickettsia australis	CP003338.1	1,418	100	16,231
Rickettsia conorii	Cell culture	Rickettsia conorii	NR_074480.2	1,421	100	6,010
Rickettsia felis	Cell culture	Rickettsia felis	CP000053.1	1,413	100	23,665
Rickettsia honei	Cell culture	Rickettsia honei	NR_025967.1	1,417	99.86	21,963
Rickettsia typhi	Cell culture	Rickettsia typhi	LS992663.1	1,420	100	39,097
*Anaplasma platys* and Mycoplasma haemocanis	Canine blood	Anaplasma platys *and* Mycoplasma haemocanis	CP046391.1, CP003199.1	1,422, 1,354	99.93, 99.79	193, 52
Anaplasma platys, Mycoplasma haemocanis, and *E. canis*	Canine blood	Anaplasma platys, Mycoplasma haemocanis, and Ehrlichia canis	CP046391.1, CP003199.1, CP025749.1	1,422, 1,354, 1,420	100, 99.79, 100	1,620, 179, 43
Filarial worm(s)						
Brugia malayi*	Canine blood	*Wolbachia* endosymbiont of Dirofilaria repens, Brugia malayi*/*Brugia pahangi, and Pseudolynchia canariensis	AJ276500.1, CP050521.1/CP034333.1, DQ115537.1	1,394, 1,414, 1,410	99.6, 99.93, 99.36	15,634, 281, 55
*Dirofilaria* sp. Hong Kong genotype*	Canine blood	*Wolbachia* endosymbiont of Brugia malayi*/*Brugia pahangi	CP050521.1/CP034333.1	1,416	99.93	1,912
Dirofilaria immitis	Canine blood	*Wolbachia* endosymbiont of Dirofilaria immitis	CP046578.1	1,421	100	520

a Classification information shows the top hit(s), identity, and length of the relevant bacterial pathogen consensus sequence, as generated by the NanoCLUST pipeline, when classified using BLASTn with the GenBank database. Asterisks indicate positive controls that had multiple microfilaria morphologies observed under the microscope, indicative of a coinfection, but the result identified via cPCR and Sanger sequencing is given. Note that results in columns 4 to 7 correspond, respectively, to the data given in column 3, i.e., the results of the NanoCLUST pipeline.

### Nanopore sequencing optimization and phylogenetic analysis.

Across all sequencing batches used to characterize the 100 canine blood DNA samples, a total of 6,950,160 raw reads were generated and then filtered by NanoCLUST to 2,641,568 utilizable reads. Details of sequencing duration, passed bases, reads pre- and postfiltering, and species richness are shown in Table 3. Batches 1 and 2 were run on one flow cell, batches 3 and 4 on a second, and batch 5 on a third, with a flow cell wash conducted between batches on the same flow cell. The sequencing duration was reduced from our first sequencing experiment on batch 1, as it was elucidated that shorter sequencing durations and less data were sufficient to detect all the bacterial pathogens found by qPCR methods. This is also reflected in the relatively consistent number of species detected (richness) per batch, despite variation in sequencing time and total data accrued.

**TABLE 3:** Nanopore sequencing data acquisition, read counts, and species richness across sequencing batches^*a*^

Batch	Sequencing time (h)	Passed bases (Mb)	Failed bases (Mb)	No. of raw reads	No. of NanoCLUST filtered reads	Mean no. of sample reads (SE)	Species richness
1	24	4,530	1,860	3,514,481	942,707	39,279 (7,338)	151
2	20.5	374	323	436,122	139,083	5,795 (989)	163
3	6	446	191	547,624	155,947	6,498 (1,746)	157
4	15	1,660	653	1,076,487	771,993	32,166 (5,858)	192
5	5	1,920	249	1,375,446	631,838	26,327 (7,858)	210
Mean		1,786	655	1,390,032	528,314	22,013	175
SE		6,74	279	499,256	145,873	6,078	10

a Passed bases are those that met a Q score of ≥8, while raw reads display these passed bases as a read count. NanoCLUST filtered reads are those that passed the NanoCLUST filtering criteria detailed in Materials and Methods, with the mean number of sample reads being the average read counts per barcode for a given batch. Species richness is given as the total number of taxonomic classifications across all 24 barcodes for each sample batch. SE, standard error.

As more data across multiple batches were collected, we identified that sequencing until ~1.5 million total raw reads (passed and failed) was sufficient to detect the common VBB at a sensitivity comparable to that of qPCR. Due to gradual pore death during sequencing, a total raw read count of ~1.5 million equated to approximately 6 h of sequencing time for the first batch run on a flow cell and 15 h for the second batch. Despite this, flow cell performance between batches was found to vary greatly due to numerous other factors, including the concentration of library loaded and whether a final PCR product size exclusion step was conducted. For batches 3 and 4, a size exclusion step using AMPure beads was included to remove PCR products of <500 bp. This was found to improve the ratio of raw to filtered reads and therefore increase the overall performance of our protocol. Nonetheless, even with interbatch variability in filtered read output, all batches performed well in comparison to the qPCR results, i.e., no batch showed a reduction in sensitivity in comparison to any other batch when benchmarked against the previously validated qPCR standard.

With the collated nanopore 16S rRNA gene data from the 100 dog samples, bacterial species that were from confirmed and suspected pathogenic species and genera were identified and extracted from the overall data set. The confirmed pathogens found were A. platys, *E. canis*, Mycoplasma haemocanis, and Bartonella clarridgeiae, while numerous suspected pathogens or bacteria of clinical importance to humans, including Brucella intermedia, Pasteurella canis, *Psychrobacter meningitidis*, and a novel sequence belonging to a hemotropic mycoplasma (NCBI accession no. ON620261), were also identified from dog blood. Table 4 shows the relevant taxonomic classification data and the number of confirmed and suspected pathogenic bacteria identified from dog blood. For the 10 samples found positive for *B. clarridgeiae* by nanopore sequencing, eight of these samples were confirmed positive by use of a *Bartonella* genus-specific qPCR (37).

**TABLE 4:** Results from NanoCLUST and BLASTn classification of confirmed and suspected bacterial pathogens from Cambodian dog blood DNA samples^*a*^

Classification	No. of samples positive	NCBI accession no.	Length (bp)	Identity (%)	Query cover
Confirmed pathogens					
Anaplasma platys	33	CP046391.1	1,461	99.73	97
Bartonella clarridgeiae	10	FN645454.1	1,405	100	100
Ehrlichia canis	29	CP025749.1	1,465	99.93	95
Mycoplasma haemocanis	20	CP003199.1	1,436	99.65	98
Uncultured *Mycoplasma* spp.*	2	KF743733.1	1,451	85.9	94
Non-VBB pathogens					
Brucella *intermedia*	6	HM030758.1	1,397	100	100
Burkholderia gladioli	17	CP068050.1	1,444	99.86	100
Haemophilus parainfluenzae	4	FQ312002.1	1,504	97.87	95
Neisseria animaloris	1	LR134516.1	1,502	99.53	96
Paracoccus yeei	1	LC371258.1	1,428	99.72	95
Pasteurella canis	2	CP085871.1	1,454	99.86	100
*Psychrobacter meningitidis*	1	AY057116.1	1,485	99.19	96

a The number of samples positive is the total number of dogs positive for the indicated pathogen out of the 100 dogs tested. The sequence indicated by an asterisk that was detected was uploaded into the NCBI GenBank database as a new entry under accession no. ON620261.

**FIG 1:**
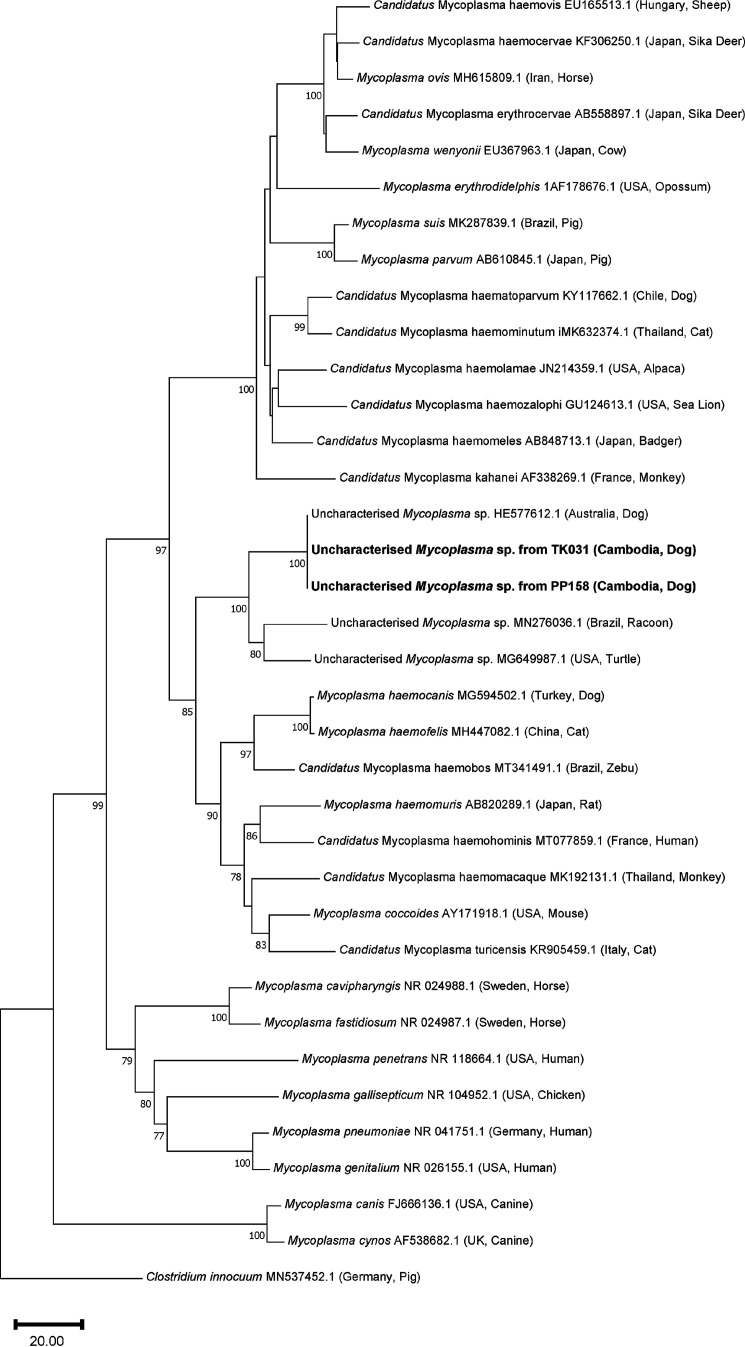
Phylogenetic relationship of the novel *Mycoplasma* taxon (bold) from the blood of two Cambodian dogs with representative sequences from across the genus *Mycoplasma* determined by the neighbor joining distance method. Relationship was based on a 528-bp stretch of the 16S rRNA gene, with bootstrap percentages greater than 75% shown and Clostridium innocuum used as an outgroup. A range of *Mycoplasma* species are shown, including hemotropic and respiratory pathogens from numerous vertebrate host species.

We used a short 528-bp stretch of the 16S rRNA gene to place our novel *Mycoplasma* sequence from two different dogs within a phylogenetic tree (Fig. 1). This sequence was homologous to an uncharacterized *Mycoplasma* 16S rRNA gene sequence 581 bp in length previously identified from the blood of free-roaming dogs from Aboriginal communities in Australia (100% identity; accession no. HE577612.1). Our novel hemotropic mycoplasma species formed a strongly supported (100% bootstrap support [bs]) sister group to uncharacterized hemotropic mycoplasmas from racoons and turtles. Additionally, this clade of uncharacterized *Mycoplasma* is, with good branch support (85% bs), adjacent to characterized hemotropic mycoplasma species found in a variety of mammals, such as *M. haemocanis*, Mycoplasma haemomuris, “*Candidatus* Mycoplasma haemohominis,” and “*Candidatus* Mycoplasma turicensis,” among others.

We also compared the full-length *Mycoplasma* sp. 16S rRNA gene consensus sequence, finding it most similar to that of an uncultured hemotropic mycoplasma found in racoons (Procyon lotor) (85.9% identity; accession no. KF743733.1) and to that of Mycoplasma haemocanis (84.96% identity; accession no. CP003199.1), thereby displaying substantial divergence from any other 16S rRNA gene entries currently in GenBank. Using these full-length 16S rRNA gene sequences, we constructed a second phylogenetic tree (1,461 bp) that had all uncharacterized *Mycoplasma* sequences omitted, as these entries typically did not contain the full-length 16S rRNA gene (see Fig. S2 in the supplemental material). This tree was largely concordant with that based on the shorter 528-bp alignment, showing that our novel sequence was most closely related to the clade of hemotropic mycoplasmas found in dogs, cats, humans, and rodents, among others (branch with 96% bs). Consensus 16S rRNA gene sequences for this novel *Mycoplasma* were identical between the two canines found positive.

### Diagnostic parameter comparison of nanopore sequencing against qPCR and Illumina sequencing.

By comparing samples that were positive by both VBB qPCR and nanopore-based metabarcoding, we were able to identify the lowest 16S rRNA gene copy number detectable by sequencing on the MinION device for the pathogens A. platys, *E. canis*, and hemotropic mycoplasmas. The highest quantification cycle (*C_q_*) values, and therefore the samples with the lowest concentrations of VBB DNA identified by our qPCR, were A. platys (*C_q_* x¯  = 32.93), *E. canis* (*C_q_* x¯  = 33.52), and hemotropic mycoplasmas (*C_q_* x¯  = 32.11). All three of these VBB-positive samples were also detected through our nanopore sequencing method, demonstrating that this method could detect 16S rRNA gene copy numbers as low as 346 in 10 μL for A. platys, 240 in 10 μL for *E. canis*, and 1,249 in 10 μL for hemotropic mycoplasmas.

To assess the performance of our newly optimized nanopore sequencing method for full-length bacterial 16S rRNA gene amplification, we compared the results of our method for the pathogens A. platys, *E. canis*, and the hemotropic mycoplasmas with those from a previously validated qPCR and an Illumina deep-sequencing methodology (12, 14). Table 5 shows the relevant kappa agreement statistics between qPCR and nanopore sequencing, indicating that for all compared pathogens, agreement was high between the two methods, with the best methodological concordance found for detection of hemotropic mycoplasmas. Disparity between methods was predominantly observed for the detection of A. platys and *E. canis*, whereby our nanopore sequencing protocol identified four samples positive for each pathogen that were not detected by qPCR. Kappa statistics between Illumina and nanopore NGS were not as concordant as those between qPCR and nanopore sequencing, although agreement for *E. canis* and hemotropic mycoplasmas was still high, with substantial agreement found for detection of A. platys (Table 6). Additionally, chi-square tests to compare the total numbers of test positives and test negatives for each pathogen found no significant differences (*P > *0.05) between any of the three tests for the three pathogens: A. platys (χ^2^ test statistic, 0.4028; *N* = 300; df 2; *P = *0.818), *E. canis* (χ^2^ test statistic, 0.7277; *N* = 300; df 2; *P = *0.695), and hemotropic mycoplasmas (χ^2^ test statistic, 0.1166; *N* = 300; df 2; *P = *0.943).

**TABLE 5:** Multiplex qPCR versus MinION sequencing agreement statistics for three bacterial blood-borne pathogens^*a*^

Pathogen	qPCR	Nanopore sequencing		Total agreement (%)	Kappa (CI)	Agreement	Kappa SE
		Neg	Pos				
A. platys	Neg	67	4	96	0.907 (0.817–0.997)	High	0.046
	Pos	0	29				
*E. canis*	Neg	71	4	96	0.899 (0.803–0.995)	High	0.049
	Pos	0	25				
Hemotropic mycoplasmas	Neg	77	0	98	0.942 (0.862–1.000)	High	0.041
	Pos	2	21				

a Pos, positive; Neg, negative; CI, confidence interval; SE, standard error. Agreement level was defined as poor if the kappa coefficient (*k*) was ≤0.20, as fair if 0.21 ≤ *k* ≤ 0.40, as moderate if 0.41 ≤ *k* ≤ 0.60, as substantial if 0.61 ≤ *k* ≤ 0.80, and as high if *k* > 0.81.

**TABLE 6:** Illumina sequencing versus MinION sequencing agreement statistics for three bacterial blood-borne pathogens^*a*^

Pathogen	Illumina sequencing	Nanopore sequencing		Total agreement (%)	Kappa (CI)	Agreement	Kappa SE
		Neg	Pos				
A. platys	Neg	63	5	91	0.795 (0.668–0.922)	Substantial	0.065
	Pos	4	28				
*E. canis*	Neg	70	6	93	0.821 (0.694–0.948)	High	0.065
	Pos	1	23				
Hemotropic mycoplasmas	Neg	76	2	95	0.852 (0.727–0.977)	High	0.064
	Pos	3	19				

a Pos, positive; Neg, negative; CI, confidence interval; SE, standard error. Agreement level was defined as poor if the kappa coefficient (*k*) was ≤0.20, as fair if 0.21 ≤ *k* ≤ 0.40, as moderate if 0.41 ≤ *k* ≤ 0.60, as substantial if 0.61 ≤ *k* ≤ 0.80, and as high if *k* > 0.81.

## DISCUSSION

Here, we demonstrate how our optimized nanopore sequencing protocol capable of sequencing the full-length 16S rRNA gene can provide species-level taxonomic resolution for the identification of a diverse spectrum of blood-borne bacteria in dogs. Furthermore, we show that our nanopore protocol can detect a range of important veterinary VBB while also having diagnostic sensitivity and specificity comparable to those of a multiplex qPCR and Illumina-based NGS method (14, 21). VBB consensus sequences for the full-length 16S rRNA gene generated by our bioinformatic pipeline consistently returned identity matches of over 99.6% against species-representative sequences from GenBank. Such results provided much finer taxonomic discernment at a species-level classification, which prior NGS methods that could only target short stretches of the 16S rRNA gene were unable to achieve without the use of confirmatory testing using a different diagnostic (21, 22, 30, 38).

The positive-control samples used throughout this study were critically informative for ascertaining whether PCR amplification biases were occurring for the detection of certain bacterial genera (Table 1) and for highlighting the levels of index cross talk or cross-contamination during library preparation (39, 40). Amplification of the ZymoBIOMICS microbial community DNA standard using the ONT barcoding primers demonstrated that although all eight of the standard’s bacteria could be detected, they were not found at the expected equal abundances. Large underrepresentation of Pseudomonas aeruginosa and overrepresentation of Staphylococcus aureus, Bacillus subtilis, and Salmonella enterica showed that the ONT primers had better binding affinity for certain bacterial species, impacting amplification efficiency and final community abundances (40). Other authors have also observed the inability or poor ability of the ONT 16S rRNA gene-targeting primers to amplify DNA from certain bacteria, such as those in *Bifidobacterium* (31, 41). Nonetheless, through our testing of canine VBB-positive controls, we could observe that even with the existence of amplification biases, there was not a significant impact on the ability of our nanopore sequencing method to detect key VBB. Additionally, our synthetic DNA positive-control construct allowed us to identify barcode index cross talk and contamination (39) while also permitting the selection of read thresholds for VBB positivity. Overall, read thresholds were either zero or low for all nanopore sequencing batches, while negative-control samples had low read counts and bacterial diversity, demonstrating very limited evidence of barcode switching and contamination.

Through consecutive nanopore sequencing batches, our library preparation method was refined, with the benefits reflected in an increase in the ratio of passed to failed bases as well as the total number of reads of ~1,500 bp in length. Key improvements included the addition of an additional cleanup and concentration step after the pooling of PCR product from all 24 samples. Here, the use of AMPure beads at a ratio of 0.55× was seen to successfully reduce the amount of the <500-bp PCR product from the library pool, an effect which substantially increased the number of ~1,500-bp reads sequenced. Moreover, the concentration of indexed library pools (60 to 80 μL) in a small quantity of eluent (~13 μL) was found to achieve a final library concentration closer to 10 ng/μL, i.e., nearer the upper target of 100 fmol recommended for library loading by ONT. Once these higher library concentrations were used, the amount of flow cell duty time dedicated to sequencing was significantly increased, with fewer nanopores in the “waiting-to-sequence” state, vastly raising the speed and amount of read data collected. In addition, through iterative running of sequencing batches, we could identify that ~1.5 million raw reads, or approximately 6 h of sequencing time on a flow cell’s first run and 15 h on its second, would generate a sequencing depth sufficient to identify VBB to a level of sensitivity comparable to that of qPCR.

Through the successive optimization of our nanopore sequencing method, we were able to develop a protocol that maximally harnessed the capabilities of long-read sequencing to characterize the blood-borne bacterial microbiome to a species-level classification. This was clearly demonstrated via the results achieved when testing our nanopore sequencing method on VBB-positive controls (Table 2). Here, closely related *Rickettsia* species were distinguishable via the use of the full-length 16S rRNA gene, a feat that would not have been achievable using one or a few hypervariable regions given the highly conserved nature of this gene across many pathogenic members of the genus (42, 43). Furthermore, through our method, reads were obtainable from a range of different sample types, including from minute quantities, such as dots of culture on slides, with genomic DNA concentrations as low as 0.05 ng/μL still detectable for Anaplasma phagocytophilum and Ehrlichia chaffeensis. Coinfections of up to three VBB from the same host were also characterizable, a feature that is particularly useful given the high prevalence of coinfected dogs observed in countries where VBB are endemic (2, 12, 35, 44).

Interestingly, some canine samples found positive for filarial worms via microscopy, cPCR, and Sanger sequencing had *Wolbachia* endosymbionts that were also detectable via nanopore sequencing of the bacterial 16S rRNA gene (Table 2). Many filaria harbor *Wolbachia* as endosymbionts, which are presumed to engage in a mutualistic relationship (45, 46). This mutualism is believed to have led to substantial coevolution between both filaria and *Wolbachia*, with shared phylogenetic splitting at similar time points between some species of each partner (45–48). Taxonomic hits from our filaria-positive samples returned hits from *Wolbachia* previously found in specific filarial worm species, with some agreement between the results of the filaria- and bacterium-specific diagnostics. Nonetheless, two of these filaria-positive controls were found to have two or more microfilaria morphologies under the microscope, indicating a coinfection and thereby complicating our ability to definitively match the bacterial nanopore results with those achieved through conventional molecular methods. Further work to explore the potential applicability of bacterial 16S rRNA gene sequencing as a proxy for diagnosis of canine filariasis would therefore be valuable.

Kappa statistics demonstrated high agreement between qPCR and nanopore sequencing for all three VBB compared (Table 5), demonstrating this method’s good diagnostic performance when evaluated against one of the most common and sensitive methods for vector-borne pathogen diagnosis (16, 49, 50). Agreement was also high in a comparison of *E. canis* and hemotropic mycoplasma using the two NGS methodologies, alongside substantial kappa agreement for A. platys as well results (Table 6). Overall, the differences in positive and negative test results gained by the three methods were not found to be statistically significant via the chi-square test (*P > *0.05), showing the high overall concordance and diagnostic performance of our new protocol. Moreover, at least 80% of the *B. clarridgeiae*-positive samples identified by nanopore sequencing were confirmed by qPCR, although there were not enough *Bartonella* positives found within our overall data set to be able to conduct a meaningful kappa agreement comparison.

When exploring disparities between the VBB qPCR and MinION results, we observed that no A. platys or *E. canis* infections were missed by nanopore sequencing, while this method detected four samples positive for each of these pathogens that were missed by qPCR (Table 5). Investigation of these eight discrepant results indicated that at least three were likely false positives, resulting from DNA carryover, as they shared a barcode with a sample that had a high read count for the same pathogen in a previous batch run on the same flow cell. ONT states that up to 0.1% of residual library may remain in a flow cell after use of their wash kits; therefore, despite the use of DNase treatment between the running of different sequencing libraries, some DNA carryover may have occurred, leading to the low read counts of some VBB-discrepant samples. For future employment of our method, to remove all risk of DNA carryover, we do not recommend the reuse of the same sample barcodes on a washed flow cell. Instead, we suggest multiplexing more barcodes onto the same flow cell and using this flow cell once, for example, by using the full suite of 96 barcodes that can be multiplexed simultaneously using kits such as EXP-PBC096 (Oxford Nanopore Technologies).

Across the 100 canine samples compared, those identified as having the lowest concentration of DNA for the pathogens A. platys, *E. canis*, and hemotropic mycoplasmas by qPCR were all detected by our nanopore sequencing protocol. This provides a good indication of our method’s analytical sensitivity, for example, by demonstrating that *E. canis* 16S rRNA gene copy numbers of at least 240 in 10 μL can be detected within the context of our pooling method and total target data. However, ascertaining specific limit-of-detection data by our method was beyond the scope of this study due to challenges of variable run performance contingent on several factors, including PCR amplification biases, number of samples multiplexed, library concentration, initial pore count, pore death rate, and total and passed reads, as well as sequencing duration, among others (51, 52).

The confirmed VBB identified via our nanopore sequencing can all inflict significant pathology on their canine hosts, while *B. clarridgeiae* is zoonotic, posing a risk to humans as well. Ehrlichia canis, for example, is a potentially fatal VBB of canines, capable of generating a multisystemic disease, while A. platys and hemotropic mycoplasma infections can exacerbate clinical signs when found concomitantly with other vector-borne pathogens (2, 53–56). In addition, a diverse range of putative pathogenic bacteria that show evidence in the literature of being opportunistic pathogens with disease-causing potential in humans were also detected by our method at read counts above the contaminant read threshold (Table 4). Environmental bacteria that can opportunistically cause infections in humans, such as Brucella intermedia (57–59), Burkholderia gladioli (60–63), Haemophilus parainfluenzae (64–68), Paracoccus yeei, and *Psychrobacter meningitidis* (69–71), were detected from our Cambodian dog blood samples. Moreover, bacterial species that may be associated with the canine oral microbiome, and etiological agents responsible for canine bite wound infections, were also identified, including Pasteurella canis (72–74) and Neisseria animaloris (75, 76). Despite these findings, due to limited data on such putative pathogenic bacteria, it cannot be unequivocally demonstrated that they were infecting the dog in which they were identified through our data alone. An alternative explanation is that these bacteria may have been introduced from the skin or the environment at the point of blood collection (21, 77, 78).

The employment of our nanopore-based sequencing method also provided us with a full-length 16S rRNA gene sequence of an uncharacterized *Mycoplasma* species detected in two canines from different regions of Cambodia. Phylogenetic (see Fig. S2 in the supplemental material) and BLASTn (Table 4) analyses demonstrated substantial divergence of this *Mycoplasma* from all other entries currently in GenBank, with only 85.9% identity to the next closest match, i.e., a *Mycoplasma* from raccoons, and 84.96% identity with the common canine-infecting species *M. haemocanis*. Given that a difference of <97% in the 16S rRNA gene is sufficient to define a new species, our data strongly support the existence of a novel *Mycoplasma* species in Cambodian dogs (79, 80). Phylogenetic placement of this species indicates that its closest relatives are many common hemotropic mycoplasmas of vertebrates, including species from dogs, cats, rodents, cattle, and humans.

Another phylogenetic tree was also constructed (Fig. 1) with a short (528-bp) stretch of the 16S rRNA gene, as this permitted phylogenetic comparison with a greater diversity of uncharacterized *Mycoplasma* sequences, due to many relevant GenBank entries not spanning the gene’s entire length. This section of sequence used for the second tree encompasses the fourth hypervariable region (V4) of the 16S rRNA gene and had 100% identity to an uncharacterized *Mycoplasma* previously found in canines from Indigenous Australian communities in Northern Australia (81). Given that a full-length 16S rRNA gene sequence is not currently available for this *Mycoplasma* found in Australian dogs (accession no. HE577612.1), it is impossible to know whether our Cambodian sequences are from the same species or if they just display homology at the V4 and surrounding stretch of the 16S rRNA gene. If they do derive from the same *Mycoplasma* species, then this would indicate the existence of a potentially uncharacterized canine-infecting *Mycoplasma* endemic to both Northern Australia and Southeast Asia, although further investigation to establish this is required.

The development and optimization of our nanopore-based bacterial metabarcoding approach shows great promise for a large array of potential future applications. Using minimal modifications, the throughput of our protocol could be made higher by the use of up to 96 unique barcodes, as has been done by Matsuo et al. (31), while its sensitivity for VBB could be augmented by using nanopore adaptive sampling, a real-time method by which sequencing can be made selective for a group of pathogens (82, 83). The highly portable nature of the MinION sequencing device highlights the potential for our method to be adapted for field use, as has been done previously during disease outbreaks in human and animal populations, e.g., for African horse sickness virus (AHSV) and Zika and Ebola viruses (84–86). In regions where canine VBB are hyperendemic, our methodology could be used to diagnose such infections in real time, substantially reducing the turnaround time for diagnosis while also providing key information for pathogen surveillance and zoonotic risks in the area (12, 87). Furthermore, our method is not inherently limited to the detection of the hemobacteriome from just canines and may be utilizable for the detection of blood-borne bacteria from humans and other mammals, while at the same time also being augmentable for the identification of other parasite groups, such as protozoa and filarial worms.

## MATERIALS AND METHODS

### Sampling and DNA extraction.

This study utilized a subset of 100 DNA samples sourced from whole blood collected from locally owned and community dogs in Cambodia as part of a previous study by Huggins et al. (12), under University of Melbourne Animal Ethics Committee permit no. 1814620.1.

In brief, following owner consent, dogs were restrained and whole blood was collected via venipuncture into EDTA tubes. Samples were temporarily kept on ice before being couriered at −20°C to the University of Melbourne, Australia, where 200 μL of thawed whole blood was extracted using the DNeasy blood and tissue kit (Qiagen, Hilden, Germany) according to the manufacturer’s protocol with a 30-min proteinase K digestion at 56°C and two final elution steps in 50 μL of total eluent. Extracted DNA was kept at −20°C until use.

All DNA extracts were quantified using a Qubit 4 fluorometer (Thermo Fisher Scientific, MA, USA) using the Thermo Fisher dsDNA HS assay kit.

### Nanopore sequencing for bacterial 16S rRNA gene metabarcoding.

Library preparation for bacterial 16S rRNA gene sequencing on the MinION Mk1B portable sequencing device (Oxford Nanopore Technologies [ONT], Oxford, UK) was conducted using the 16S barcoding kit 1-24 (SQK-16S024) and the “rapid sequencing amplicons—16S barcoding protocol” version 16S_9086_v1_revU_14Aug2019 with some minor modifications. Key changes to this protocol were adopted to improve amplification of 16S rRNA gene bacterial DNA from low-input DNA samples such as blood. Quality control of input genomic DNA from blood was conducted using a NanoDrop ND-1000 spectrophotometer (Thermo Fisher Scientific) to ensure that 260/280 and 260/230 ratios were optimal for use in sequencing. As described in the ONT protocol, 25 μL of LongAmp hot start *Taq* 2× master mix (New England Biolabs, MA, USA) was mixed with 5 μL of Ambion nuclease-free water (Life Technologies, CA, USA), 10 μL of genomic blood extracted DNA from dogs (irrespective of starting DNA concentration), and 10 μL of a 16S barcode. These barcodes use the 27F (5′-AGAGTTTGATCMTGGCTCAG-3′) and 1492R (5′-CGGTTACCTTGTTACGACTT-3′) primers to amplify the full-length 16S rRNA bacterial gene while also containing one of 24 unique molecular identifiers to permit multiplexing of up to 24 samples simultaneously. PCR was then conducted on a T100 thermal cycler (Bio-Rad, CA, USA) using the following conditions: 1 cycle of 95°C for 1 min, 40 cycles of 95°C for 20 s, 55°C for 30 s, and 65°C for 2 min, and a final extension of 65°C for 5 min. Different separate physical containment areas were utilized for DNA extraction, pre-PCR, and post-PCR experiments, with all PCRs performed in a PCR hood under sterile conditions with filter tips following UV sterilization of the workspace.

PCR product was cleaned using a 1× ratio of AMPure beads (Beckman Coulter, CA, USA) with a 15-min incubation on a HulaMixer (Thermo Fisher Scientific) and two washes with freshly made 70% ethanol, followed by a final elution in 10 μL of 10 mM Tris-HCl, pH 8.0, with 50 mM NaCl. Correct amplification of the expected ~1,500-bp product was assessed using a 4200 TapeStation system (Agilent Technologies, CA, USA), and the final DNA concentration was analyzed using a Qubit fluorometer. Cleaned amplicons were then mixed in approximately equimolar ratios to a final volume of 100 μL under the condition that when a sample’s DNA concentration was too low to achieve equimolarity, the maximum amount of DNA was added to the pool. Pools were then quantified on a Qubit fluorometer with average DNA concentrations of approximately 2 to 3 ng/μL. A target of ~100 fmol in 10 μL is required for optimal sequencing performance; hence, a final AMPure bead cleanup and concentration step was used whereby ~60 μL of pool was incubated with a 0.55× ratio of AMPure beads for 15 min on a HulaMixer, followed by two washes in 70% ethanol and a final elution in 13 μL of 10 mM Tris-HCl, pH 8.0, with 50 mM NaCl. A 0.55× ratio of AMPure beads was required to size exclude any product of <500 bp, with the outcome of this checked using a final TapeStation assessment of the cleaned and concentrated pool. This amplicon pool (prepared library) then had 1 μL of rapid adapter (RAP) added to it for a 5-min incubation step. The flow cell was then prepared for sequencing as per the standard ONT protocol with the library pool finally loaded and sequencing commenced.

Samples were run in batches of 24, with each batch including a negative DNA extraction control and a positive control consisting of a uniquely identifiable 1,480-bp gBlock synthetic DNA strand (Integrated DNA Technologies, IA, USA) of the 16S rRNA gene from Aliivibrio fischeri as previously reported by Huggins et al. (20) (see the design in Text S1 in the supplemental material). For the first batch of samples prepared for sequencing, a diluted ZymoBIOMICS microbial community DNA standard (Zymo Research, CA, USA) was used to assess PCR amplification biases and a PCR negative control containing Ambion water was run to detect PCR reagent contaminants. A total of six batches of 24 samples were run across three different R9.4.1 flow cells (ONT) to accommodate all 100 dog blood samples as well as positive and negative controls. Each flow cell was used to run two different libraries with the flow cell wash kit (ONT) used between runs to minimize DNA carryover.

To confirm that a diverse range of blood-borne bacteria of veterinary importance could be detected by our method, we tested it on a range of positive-control samples from blood and culture, previously characterized by conventional PCR and Illumina sequencing (Huggins et al. [12, 21, 38]). DNA of positive controls sourced from bacterial culture were extracted using the DNeasy blood and tissue kit (Qiagen, Hilden, Germany), as described for whole blood. Species of positive-control DNA tested are listed in Table 2.

Nanopore sequencing used a MinION Mk1B device was conducted on a Legion 7i Gen 6 laptop (Lenovo, Quarry Bay, Hong Kong) that utilizes a NVIDIA GeForce RTX 3070 (8 GB) graphics processing unit (GPU) and an 11th-generation Intel Core i7-11800H (8C) processor to permit local base-calling with Guppy. Sequencing was initiated through MinKNOW version 21.10.4 with fast base-calling and a Q score of ≥ 8 for between 5 and 25 h, depending on the amount of data required. Once sequencing was stopped, FAST5 reads were base called using the super-high-accuracy base-calling model with barcode removal with using Guppy version 5.0.17.

### Bioinformatics.

Given the relatively high error rate of the ONT R9.4.1 flow cell (29, 88) a variety of bioinformatic processing pipelines were tested on the data accrued from sequencing of genomic DNA positive controls of known composition as well as artificial mock communities of these controls. Positive-control species composition had been previously characterized using Illumina-based deep sequencing and conventional PCR (14). Two pipelines were chosen for processing and taxonomic classification of FASTQ data generated: Emu (29) and NanoCLUST (28).

Initial analysis was conducted using Emu (29), which uses an expectation-maximization algorithm to classify all inputted reads and rapidly generate taxonomic abundances alongside read counts for each classification using the Ribosomal Database Project (RDP) release 11 database. This pipeline was useful for gaining a quick assessment of all bacteria present in a sample, but it was also observed to overinflate the diversity of some samples. For example, Emu occasionally categorized a proportion of sequences as belonging to a different species of the same genus from that which was known to be present in a given positive control. Next, the same FASTQ data were processed using NanoCLUST (28), a bioinformatic pipeline which conducts multiple quality control, read clustering, polishing, and consensus-forming steps, followed by classification of consensus sequences with the RDP database. Optimal parameters for NanoCLUST were found to be a minimum read length of 1,200 bp, a maximum read length of 1,700 bp, a minimum cluster size of 30, and 100 reads used for polishing. NanoCLUST was not found to overinflate taxonomic diversity in positive controls, and if organisms were missed by NanoCLUST but identified by Emu, then the NanoCLUST pipeline was rerun using a lower cluster size of 10 to improve sensitivity. Read counts and classifications generated by NanoCLUST were taken as the final data set used for comparison and validation against qPCR results. NanoCLUST also generates consensus sequences utilizable for further downstream analyses. Read thresholds to determine whether a sample is positive for a given bacterial pathogen were calculated as described by Huggins et al. (12). Briefly, reads of the uniquely identifiable A. fischeri positive-control sequence that was found in samples other than the positive control and reads from known VBB found in negative controls were used to inform the read cutoff threshold for a given library. The appearance of such sequences in negative controls or canine samples may be due to occasional index misreading, sequencing error, chimeric reads, or low-level cross-contamination during library preparation (39, 89). The read threshold for a given library was the highest read count of a positive-control sequence in a nonpositive control sample or VBB count in a negative-control sample, whichever was larger. Using this method, read thresholds were found to be low at between 0 to 144 reads for all batches. If pathogen reads from a blood sample were found to be lower than the batch’s threshold, then the sample was defined as negative for that particular pathogen, i.e., a false positive.

### Methodological comparison with qPCR and Illumina sequencing.

The same 100 canine blood DNA samples that underwent nanopore sequencing, as well as the relevant extraction reagent negative controls, were also analyzed in duplicate using a previously developed and validated multiplex qPCR for the canine VBB A. platys, *E. canis*, and hemotropic mycoplasmas (14). Additionally, these same samples had also been previously characterized by an Illumina sequencing metabarcoding method with the full protocol reported by Huggins et al. (12), thereby providing another data set against which to benchmark the diagnostic parameters of the nanopore sequencing protocol.

Serial dilutions of gBlock synthetic DNA targets (IDT) for the three pathogens targeted by the canine VBB qPCR were produced as described by Huggins et al. (14) so that standard curves could be generated, allowing the conversion of *C_q_* values into an estimated DNA concentration for a given pathogen by use of the equation found from the line of best fit. Therefore, samples found positive by both qPCR and nanopore sequencing could have their *C_q_* value converted into a DNA concentration and then copy number by the following formula:

$$\text{number of copies}=\frac{\text{amount of DNA }\left(\text{ng}\right)\;\times \;6.022\;\times \;10^{23}\;}{\text{amplicon length }\left(\text{bp}\right)\;\times \;10^{9}\;\times \;660}$$

Identification of the lowest number of copies detected by our nanopore sequencing method across our data set could thereby provide us with an indication of our assay’s sensitivity, with such comparisons of relative sensitivity to that of a qPCR assay having been conducted before (90). Additionally, a *Bartonella*-specific transfer mRNA (*ssrA*)-targeting singleplex qPCR (37) was employed to conduct confirmatory assessments of samples found to be *Bartonella* positive via nanopore sequencing.

Kappa statistics were used to compare the agreement of nanopore sequencing positivity for A. platys, *E. canis*, and hemotropic mycoplasmas against the results from the canine VBB qPCR (14) and the Illumina sequencing results (12) in SPSS Statistics 28 (IBM, New York, USA). We used the formula defined by McHugh (91), with the agreement between tests considered poor if *k* ≤ 0.20, fair if 0.21 ≤ *k* ≤ 0.40, moderate if 0.41 ≤ *k* ≤ 0.60, substantial if 0.61 ≤ *k* ≤ 0.80, and high if *k* > 0.81 (91). Furthermore, 3 × 2 chi-square test tables were used for A. platys, *E. canis*, and hemotropic mycoplasmas to assess whether there was a statistically significant difference between the counts of test positives and negatives between the three different methodologies compared.

### Phylogenetic analysis.

Phylogenetic analysis was conducted on the novel hemotropic mycoplasma sequences detected using relevant 16S rRNA gene sequences sourced from GenBank and aligned to our consensus sequence in Mega version 11.0.11. Analysis was carried out using the neighbor-joining (NJ) distance method (92), with evolutionary distances computed using the “number of differences” method (93), including “transitions and transversions” for the nucleotide data. The rates of evolution among sites were considered uniform, and gaps were treated using pairwise deletion. Overall, 2,000 bootstrap replicates were performed and are reported as bootstrap support percentages (bs), with any percentages lower than 75% omitted. Clostridium innocuum was used as an outgroup for all phylogenetic analyses. Two phylogenetic trees were made, one using a smaller 528-bp stretch of the 16S rRNA gene and another using the full-length 16S rRNA gene (1,461 bp) found in Fig. S2. The tree using a smaller region of this gene had a greater number of uncharacterized but closely related hemotropic mycoplasma sequences that could be directly compared, whereas many of these sequences did not have a full-length 16S rRNA gene entry and were therefore omitted from the full-length tree.

### Ethical approval.

This study was approved by the Office of Research Integrity and Ethics, University of Melbourne, under ethics permit no. 1814620.

### Data availability.

All nanopore-based NGS data produced in the present study are available from the NCBI BioProject database under BioProject ID no. PRJNA601241 and BioSample ID no. SAMN13870772 to SAMN18323751, specifically Sequence Read Archive (SRA) accession no. SRR19370842 to SRR19370953.
