# Supplementary material for: Nanopore Sequencing Using the Full-Length 16S rRNA Gene for Detection of Blood-Borne Bacteria in Dogs Reveals a Novel Species of Hemotropic Mycoplasma
Source: Microbiol Spectr. 2022 Oct 17;10(6):e03088-22. doi: 10.1128/spectrum.03088-22 (PMC9769565; doi:10.1128/spectrum.03088-22)
Supplement: Supplemental file 1 — Supplemental material. Download spectrum.03088-22-s0001.pdf, PDF file, 0.4 MB [file spectrum.03088-22-s0001.pdf]

Supplementary Information 1: Sequence of our unique positive control gBlock DNA construct used as a positive control for full-length bacterial 16S rRNA metabarcoding analysis.

Positive control sequence is comprised of 27F and 1429R primer binding sites (underlined) and the full-length 16S rRNA gene of *Aliivibrio fischeri* with this gBlock construct synthesised by Integrated DNA Technologies (Iowa, USA). The degenerate M base in the 27F primer was changed to an A (bold), total gBlock sequence length is 1,480 bp.

5’ -

AGAGTTTGATC**A**TGGCTCAGATTGAACGCTGGCGGCAGGCCTAACACATGCAAGTCGAGCGGAAACGACTTAACT
GAACCTTCGGGGAACGTTAAGGGCGTCGAGCGGCGGACGGGTGAGTAATGCCTGGGAATATGCCTTAGTGTGGG
GGATAACTATTGGAAACGATAGCTAATACCGCATAATGTCTTCGGACCAAAGAGGGGGACCTTCGGGCCTCTCGC
GCTAAGATTAGCCCAGGTGAGATTAGCTAGTTGGTGAGGTAAGAGCTCACCAAGGCGACGATCTCTAGCTGGTCT
GAGAGGATGATCAGCCACACTGGAACTGAGACACGGTCCAGACTCCTACGGGAGGCAGCAGTGGGGAATATTGC
ACAATGGGCGAAAGCCTGATGCAGCCATGCCGCGTGTATGAAGAAGGCCTTCGGGTTGTAAAGTACTTTCAGTAG
GGAGGAAGGTGTTGTAGTTAATAGCTGCAGCATTTGACGTTACCTACAGAAGAAGCACCGGCTAACTCCGTGCCA
GCAGCCGCGGTAATACGGAGGGTGCGAGCGTTAATCGGAATTACTGGGCGTAAAGCGCATGCAGGTGGTTCATT
AAGTCAGATGTGAAAGCCCGGGGCTCAACCTCGGAACCGCATTTGAAACTGGTGAACTAGAGTGCTGTAGAGGG
GGGTAGAATTTCAGGTGTAGCGGTGAAATGCGTAGAGATCTGAAGGAATACCAGTGGCGAAGGCGGCCCCCTGG
ACAGACACTGACACTCAGATGCGAAAGCGTGGGGAGCAAACAGGATTAGATACCCTGGTAGTCCACGCCGTAAA
CGATGTCTACTTGGAGGTTGTTCCCTTGAGGAGTGGCTTTCGGAGCTAACGCGTTAAGTAGACCGCCTGGGGAGT
ACGGTCGCAAGATTAAAACTCAAATGAATTGACGGGGGCCCGCACAAGCGGTGGAGCATGTGGTTTAATTCGATG
CAACGCGAAGAACCTTACCTACTCTTGACATCCAGAGAATTCGCTAGAGATAGCTTAGTGCCTTCGGGAACTCTGA
GACAGGTGCTGCATGGCTGTCGTCAGCTCGTGTTGTGAAATGTTGGGTTAAGTCCCGCAACGAGCGCAACCCTTAT
CCTTGTTTGCCAGCACGTAATGGTGGGAACTCCAGGGAGACTGCCGGTGATAAACCGGAGGAAGGTGGGGACGA
CGTCAAGTCATCATGGCCCTTACGAGTAGGGCTACACACGTGCTACAATGGCGCATACAGAGGGCTGCAAGCTAG
CGATAGTGAGCGAATCCCAAAAAGTGCGTCGTAGTCCGGATTGGAGTCTGCAACTCGACTCCATGAAGTCGGAAT
CGCTAGTAATCGTAGATCAGAATGCTACGGTGAATACGTTCCCGGGCCTTGTACACACCGCCCGTCACACCATGGG
AGTGGGCTGCAAAAGAAGTGGGTAGTTTAACCTTCGGGAAAGTCGTAACAAGGTAACCG **– 3’**

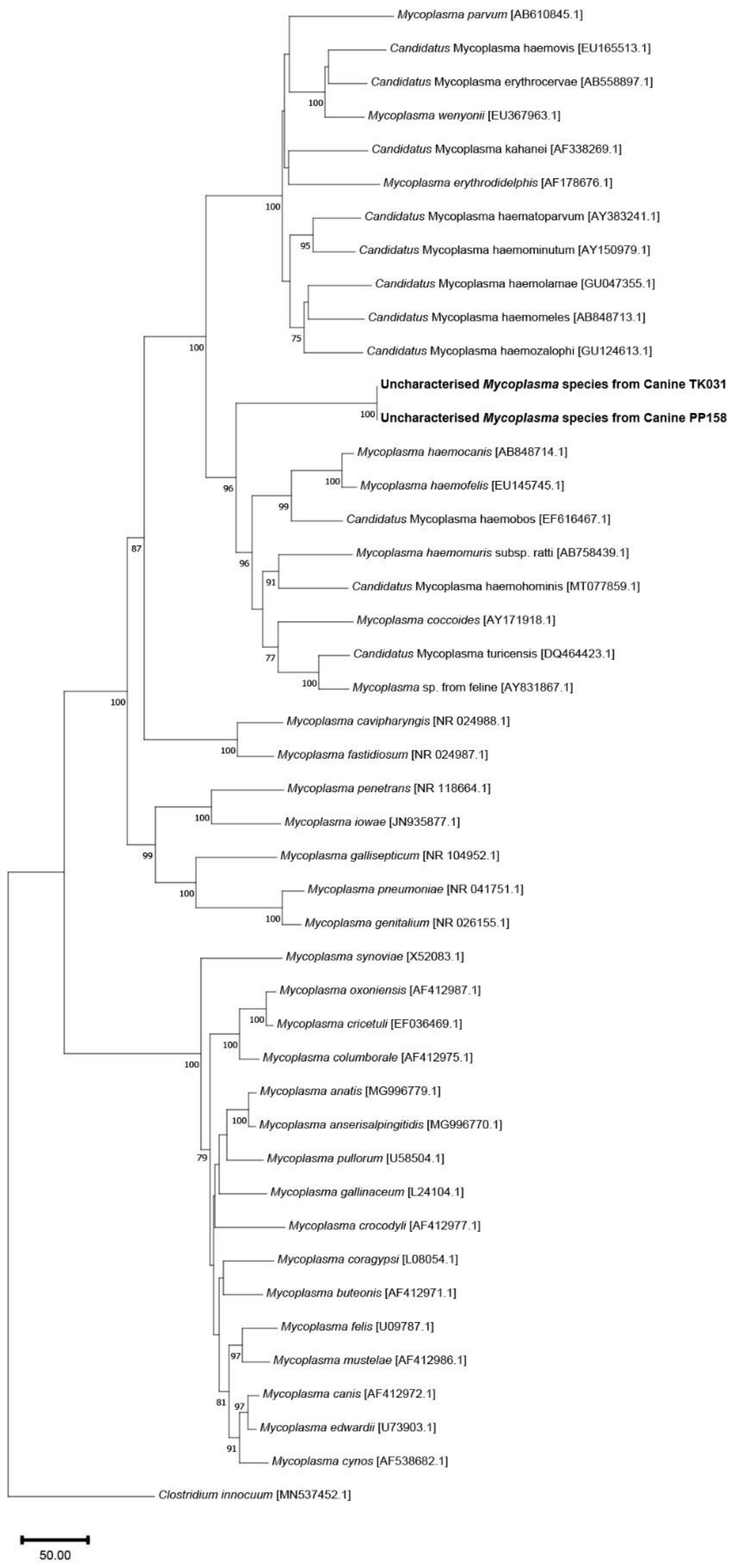

Supplementary Information 2: Phylogenetic relationship of the novel *Mycoplasma* taxon (in bold, NCBI accession ON620261) from the blood of two Cambodian dogs alongside representative sequences from across the genus *Mycoplasma* employing the neighbour-joining distance method. Relationship based on the full-length 16S rRNA gene (1,461 bp), with bootstrap percentages greater than 75% shown and *Clostridium innocuum* used as an outgroup.
